# A State-of-Art Review on Multi-Drug Resistant Pathogens in Foods of Animal Origin: Risk Factors and Mitigation Strategies

**DOI:** 10.3389/fmicb.2019.02091

**Published:** 2019-09-06

**Authors:** Fernando Pérez-Rodríguez, Birce Mercanoglu Taban

**Affiliations:** ^1^Department of Food Science and Technology, Faculty of Veterinary, Agrifood Campus of International Excellence (ceiA3), University of Córdoba, Córdoba, Spain; ^2^Dairy Technology Department, Faculty of Agriculture, Diskapi Campus of Veterinary and Agriculture, Ankara University, Ankara, Turkey

**Keywords:** antimicrobial resistance, bacteriocin, biocontrol, lactic acid bacteria, food safety, food-related environment, microbial interaction, risk assessment

## Abstract

Most of the foodborne microbial diseases are linked to foods of animal origin such as milk, meat, and poultry. Nowadays, the presence of multi-drug resistant (MDR) pathogens in foods is becoming an increasingly public health concern worldwide due to the overuse of antimicrobial drugs in animal feed. MDR pathogens can enter the food chain by posing a significant risk to both animals and consumers. MDR pathogens causing infections are untreatable due to their resistance to various antibiotics, primarily cephalosporin and carbapenems and to their extended-spectrum beta-lactamase (ESBL)-producing capability. In addition, foods of animal origin and food-related environments can be likely vehicles for spreading of multi-drug resistance genes, which accelerates the thriving of global antibiotic resistance. This paper reviews the role of foods of animal origin as a vehicle for MDR pathogens, stressing the contribution of food processes, environments, and storage conditions in dissemination and reduction of antimicrobial resistances (AMRs). Controlling the growth of MDR microorganisms and limiting the transmission/expression of AMR genes in food ecosystems could be an effective mitigation strategy, putting the focus on food processes as a part of the solution for AMR in foods. Bioprotective cultures are also a promising and environmentally friendly technology to reduce the incidence of MDR pathogens, though caution is taken as microbial starters and probiotics can also carry AMR. Finally, applying Whole Genome Sequencing (WGS) and predictive microbiology, within a Risk Assessment framework, is key to get insight into those mechanisms and conditions along the food chain favoring or reducing AMR.

## Introduction

The emergence of multi-drug resistant (MDR) pathogens has been one of the most critical public health problems in the last decades. In addition to occurrence of nosocomial infections with high mortality rates due to the dissemination of ESKAPE pathogens [*Enterococcus* (*E*.) *faecium*, *Staphylococcus* (*S*.) *aureus*, *Klebsiella* (*K*.) *pneumoniae*, *Acinetobacter* (*A*.) *baumannii*, *Pseudomonas* (*P*.) *aeruginosa*, and *Enterobacter* species which can “escape” from the biocidal action of multiple types or classes of antibiotics] both in humans and animals (Rice, 2008; Pendleton et al., 2013; Santajit and Indrawattana, 2016), more recent challenges such as mobile colistin-resistant (mcr) strains and New Delhi metallo-β-lactamase-1 (NDM-1)-producing strains in food-producing animals (FPA) are emerging as reservoirs of colistin resistance and β-lactam antibiotics including the last-resort antibiotic carbapenem resistance respectively in the public nowadays (Al-Tawfiq et al., 2017; Khan et al., 2017; Ghafur et al., 2019). The incidence of these pathogens varies based on the bacterial species, antimicrobial groups, and the most of all the geographical location in the world. According to the Centers for Disease Control and Prevention (CDC) report in 2013, at least 23,000 people died from more than 2 million people infected with antibiotic-resistant bacteria each year in the USA and the etiological agents in those rapidly increasing rates of infections were mostly found as methicillin-resistant *S. aureus* (MRSA), vancomycin-resistant *E. faecium* (VRE), and fluoroquinolone-resistant *P. aeruginosa* (Centers for Disease Control, 2004, 2013). Moreover, data of European Antimicrobial Resistance Surveillance Network (EARS-Net) showed that high levels of antibiotic resistance had still remained in the European Union (EU) for several bacterial species, and their percentages were generally higher in southern and south-eastern Europe than in northern Europe between the years of 2014 and 2017. As an example, more than one-third of the *K. pneumoniae* were reported as resistant to at least one of the antibiotic groups of aminoglycosides, cephalosporins, fluoroquinolones, and carbapenems. Besides, combined resistance of *Escherichia* (*E.*) *coli* and *K. pneumoniae* to several antimicrobial groups by production of extended-spectrum beta-lactamase (ESBL) was also commonly determined. While carbapenem resistance percentage of *K. pneumoniae* was reported as almost 10%, it was at higher percentages for *A*. *baumannii* and *P. aeruginosa*, including a significant increase from 10.4% in 2014 to 14.9% in 2017 for vancomycin-resistant *E. faecium*. Likewise, prevalence of plasmid-borne *mcr-1* and *bla_NDM_* genes particularly in *E. coli* strains has undermined the antimicrobial effectiveness of colistin and carbapenems. This has resulted in treatment failures due to a lack of effective therapeutic alternatives for microbial diseases. On the contrary, although the resistance situation of MRSA isolates appeared to continue, a decrease from 19.6% in 2014 to 16.9% in 2017 was reported (EARS-Net, 2018).

The presence of the MDR pathogens in foods of animal origin such as milk, meat, and poultry has dramatically increased over the last few years. More important is their ability in evolving to gain novel characteristics, particularly multi-drug resistance. These recently emerging MDR pathogens were previously unknown to the animal food industry since only few reports on their presence in foods of animal origin were available, but the epidemiological circumstances have changed with the advent and spread of them in foods of animal origin due to the overuse of antimicrobial drugs in FPA; thus, MDR zoonotic bacteria are able to reach the intestinal tract of humans (Barber et al., 2003; Muloi et al., 2018). Therefore, although there is an acceptation that the transmission of MDR pathogens primarily occurs from infected person to other persons, highly resistant bacterial infections are no longer limited to hospital-acquired infections since foods of animal origin are frequently contaminated with MDR pathogens and, hence, has started to become the possible source for the exposure of not only high-risk groups like vulnerable patients, but also whole public (Lee, 2003; Centers for Disease Control, 2013).

The awareness of the potential risks of antimicrobial resistance (AMR) due to the severity of the diseases that MDR zoonotic pathogens may cause is a growing concern for food industry, which could lead to a loss of consumer confidence and accordingly a fall in demand of foods of animal origin. From this perspective, the increasing number of MDR pathogens in foods of animal origin not only imposes a significant burden on the global food industry, but also causes significant but avoidable economic losses. Therefore, there is an urgent need for a better understanding of the risk factors along the food chain. This review has been designed to shed light on the role of foods of animal origin as a vehicle for bacteria-specific antibiotic resistance, including the possible contamination routes of MDR pathogens and proposing the Risk Assessment (RA) framework as basis for the study. The review is also focused on the mitigation strategies such as the possibility of use of bioprotective cultures to prevent and/or control the incidence of MDR pathogens in foods and hence to combat this growing threat through reducing pathogen growth and limiting dissemination of antibacterial resistance genes in the food environment.

## The Role of Foods of Animal Origin on Multi-Drug Resistance Diseases and Risk Factors

**Figure 1:**
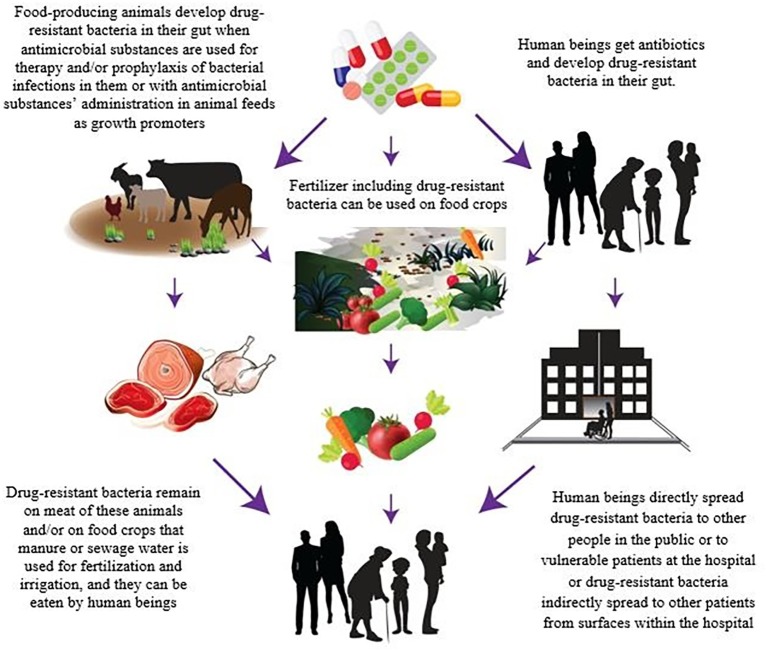
Antibiotic resistance spread through foods.

In order to control the dissemination of MDR diseases through the foods of animal origin, the sources of contamination must be identified. There are several complex routes of transmission of these resistant bacteria and/or AMR genes along the food chain. However, the relative contribution of foods to the global burden of infections caused by MDR pathogens has not been estimated yet (Likotrafiti et al., 2018). Animal production and aquaculture are known as the primary sectors where the large majority of the antibiotics are used (Verraes et al., 2013). The overuse or misapplication of antimicrobial drugs for therapy and prophylaxis of bacterial infections in FPA or with their use in animal feeds as growth promoters in food animal production are leading to the development of MDR in pathogens during livestock production (Figure 1; Barber et al., 2003; Aarestrup, 2005; Normanno et al., 2007; Verraes et al., 2013; Schrijver et al., 2018). van Boeckel et al. (2015) presented the first global map of antibiotic consumption in livestock and provided a baseline estimate of its global importance in the future. It is also now generally accepted that more antibiotics are used in FPA than the amount administered in humans (Centers for Disease Control, 2013). This has unavoidably led to the emergence of resistant pathogenic bacteria in animals’ gut, and because of their resistance to commonly used therapeutic antibiotics, these bacteria may cause infections for which there are limited therapeutic options. In other words, every time that the antibiotic, which is not needed, is administered to FPA, it creates a risk of development of a resistant infection in humans in the near future (Smith et al., 2002; Silbergeld et al., 2008; Callens et al., 2018). Besides that, MDR pathogens can also reach crops and plants through contaminated manure or sewage water that is used for fertilization and irrigation (Figure 1; Nilsson, 2012; Centers for Disease Control, 2013; Pruden et al., 2013; Durso and Cook, 2014). In this respect, a recent report published by the European Food Safety Authority (EFSA) and European Center for Disease Prevention and Control (ECDC) presented scientific evidence of the existence of a linkage between the use of antibiotics in livestock production and AMR in foodborne pathogens (European Food Safety Authority/European Centre for Disease Prevention, 2018). It was also demonstrated that the use of third- and fourth-generation cephalosporins to treat *E. coli* infections in livestock was related to resistances of *E. coli* found in humans. In addition, ciprofloxacin-resistant *Salmonella*, and macrolides and fluoroquinolones-resistant *Campylobacter* strains are on the rise in FPA (Kumar et al., 2012; Mukherjee et al., 2013).

### The Impact of the Food Chain on the Antimicrobial Resistance Transmissions

The food chain is one of the main driving forces in the transmission of MDR due to the fact that foods are not sterile and usually present microbiological contamination or can become contaminated at specific stages along the food chain (i.e., cross contamination, recontamination) (Cahill et al., 2017). Indeed, the food chain can act as a booster for MDR dissemination, allowing for survival or even increase of MDR pathogens. MDR transmission is not exclusive for food of animal origin or fish, and plant foods may also harbor MDR microorganisms as vegetables can be contaminated during primary production, through water contaminated with fecal material from effluent of surrounding farms. In general, pathogens are not frequent in foods, and the major risk arises from the high prevalence of non-pathogenic microorganisms that can transfer AMR genes to other microbial species, including foodborne pathogens. It is well documented that genes encoding resistance are transmissible between different bacteria in FPA and also from them to bacteria in foods and in humans by horizontal gene transfer (i.e., transformation, conjugation, and transduction) (Aarestrup, 2005; Appelbaum, 2006; Hammerum et al., 2010; Verraes et al., 2013). In a study, identical gentamicin resistance gene was found in *Enterococcus* spp. isolated from FPA, retail foods, and humans from geographically different areas (Donabedian et al., 2003) which can also be accepted as a supporting result of the dissemination of gentamicin-resistant *Enterococcus* spp. from FPA to humans *via* the food chain. Liu et al. (2019) also showed that mobile elements harboring *mcr-1* and *bla_NDM_* acquired by FPA strains are the ways of their transmission to our foods and then from foods to humans, after finding structurally similar *mcr-1* and *bla_NDM_* bearing plasmids both in foods and in clinical isolates. In addition, identical *mcr-1* genes were reported in 21% healthy swine at slaughter, 15% marketed pork and chicken meat, and 1% patients in China in 2016 (Yi-Yun et al., 2016). On the other hand, AMR gene transfer is not only specific to live cells; conversely, stressed or partially inactivated cells are able to confer resistance to other microorganisms, including pathogens, or microbiota in general and, after ingestion, mobilized to intestinal microbiota in humans through the gene transfer mentioned above (Verraes et al., 2013).

Notwithstanding the foregoing, we should not narrow the origin of drug resistances to primary production as the food chain can generate resistances by itself. The pressure exerted by the wide use of biocides for food production such as disinfectants, preservatives, and other chemicals or even environmental and process conditions applied through the food distribution chain has been proved to trigger the adaption of microbial populations by developing transient resistances (Capita and Alonso-Calleja, 2013; Händel et al., 2013).

### Multi-Drug Resistant Foodborne Pathogens From Farm to Fork

The transmission routes of MDR foodborne pathogens along the food chain and how they can reach consumers are not clear at present. The relevance of the mechanisms mentioned above with respect to the possibility that resistant pathogens can be originated at primary production and spread through the food chain is also unknown. However, it is reasonable to consider that MDR pathogens from a very early food stage (i.e., primary production) can enter and remain in the food systems and (re)contaminate, survive, and/or grow in food or food environments resulting in their presence of both raw food and ready-to-eat products at the consumption stage (Figure 1; Lee, 2003; Verraes et al., 2013). Although the use of preservation technologies and/or food-processing technologies (i.e., hurdle technology concept) can play a similar role to that found for non-resistant pathogens by inactivating populations, and reducing risk (Mathur and Singh, 2005; Lester et al., 2006; Verraes et al., 2013), it has been also suggested that the sublethal conditions induced by preservation methods can stimulate the horizontal transmission of plasmids containing AMR genes (McMahon et al., 2007). Even the complete elimination of bacteria by lethal treatments does not assure that AMR is not transmitted, since DNA released from lysed cells can still be transferred, by the process of transformation to living microorganisms (pathogenic or commensal) on foods or in human digestive system. In addition, environmental stress produced by these technologies can drive pathogens to adapt to the stressful environment by evoking the expression of the resistance genes, and, as a consequence, enhanced resistance capacity and changes in virulence and infectivity of the populations (Horn and Bhunia, 2018). In spite of the few studies carried out hitherto, it has been proved that sublethal stress produced by thermal, acidic, and saline conditions can affect the phenotypic resistance (Verraes et al., 2013). For example, there are data suggesting that sublethal high temperatures can reduce the presence of phenotypic resistance while increase in salt concentrations or reduction of pH is rather related to its increase (McMahon et al., 2007).

### Lactic Acid Bacteria as Reservoirs of Antimicrobial Resistance

The fermentative processes carried out in foods such as technological processes or those occurring naturally are also a key aspect in the transmission of resistances. Lactic acid bacteria (LAB), one of the most common microbiota in foods, and widely used as starters for specific food products (yoghurt, cheese, dry-cured meat, etc.) can also act as reservoirs of AMR genes similar to those found in clinical pathogens and hence cause a spread of resistance genes to foodborne pathogenic bacteria (van den Bogaard and Stobberingh, 2000; Smith et al., 2002). As an example, identical tetracycline-, erythromycin-, and vancomycin-resistant genes that were found in clinical bacterial species were also detected in *Lactococcus lactis* and *Lactobacillus* species isolated from fermented meat and milk products (Mathur and Singh, 2005). Besides, some human commensal bacteria revealed the presence of AMR determinants within their genomes and therefore they show intrinsic AMR (Cox and Wright, 2013). Among the LAB isolated from fermented foods, transferable resistance genes were rare (European Food Safety Authority, 2008) and mostly *Enterococcus* spp. was found to have antibiotic resistance, especially to vancomycin although resistance to chloramphenicol and erythromycin was also observed (Teuber et al., 1999). Therefore, the presence of AMR genes in starter, adjunct, and/or probiotic cultures that are intentionally added during animal food processing can also pose a substantial risk for increasing foodborne diseases that cannot be treated by current antibiotics (Verraes et al., 2013).

## Moving Toward a Quantitative Risk Assessment Approach to Develop a Better Control of Multi-Drug Resistant Along the Food Chain

Risk Assessment is a structured and science-based method intended to estimate human risk associated with exposure to foodborne hazards (Codex Alimentarius Commission, 2014). Microbiological Risk Assessment (MRA) has been widely used to estimate risk and risk factors linked to microbiological hazards along the food chain, being a pillar in the development of food policy and control measures for the most relevant foodborne pathogens (World Health Organization/Food and Agriculture Organization, 2002, 2016; Pérez-Rodríguez et al., 2017). The application of MRA in the context of MDR foodborne pathogens is needed more than before in order to shed light on the main transmission routes of MDR and identify the relevant factors along the food chain (Claycamp and Hooberman, 2004; Geenen et al., 2010). By using a Risk Analysis framework, efficient mitigation strategies for antimicrobial resistant microorganisms could be developed and implemented to fight against resistant foodborne pathogens. One of the most critical elements in MRA is the generation of data that allow to accomplish all MRA steps properly. For that, the development of surveillance programs that can incorporate reliable data on the presence of resistance microorganisms and genes throughout the food chain is a primary requirement. Whole Genome Sequencing (WGS) analysis is proposed as a key tool to accomplish this task, providing data on complex and non-culturable communities in addition to providing an information basis to determine the linkage between MDR microorganisms in foods and observed foodborne disease cases. On the other hand, the interpretation of genomic data into the current MRA paradigm is complex, but MRA is estimated to move to the study of microbial behavior (i.e., expression of genes) rather than the taxonomic and genotypic identification (Cocolin et al., 2018). Therefore, incorporation of studies of predictive microbiology (Pérez-Rodríguez, 2013), based on the mathematical characterization of microbial response in ecofood systems, becomes an innovative discipline that may be applied to better elucidate the main transmission routes and food process parameters contributing to MDR dissemination. In this sense, it is also reported that antimicrobial resistant mutants can show a different phenotype than wild microorganisms due the metabolic cost produced by the mutations (Schulz zur Wiesch et al., 2010; Händel et al., 2013). This fact leads us to formulate the hypothesis that if there are some food process parameters and conditions, or the combination of them, more affecting the viability and spread of MDR microorganisms, maybe, this information could be exploited to develop more effective control measures to minimize their transmission along the food chain.

## Mitigation Strategies to Combat Growing Threat of Multi-Drug Resistant Pathogens in Foods

The presence of MDR pathogens in foods of animal origin could safe food production in future. Taking an *One Health* perspective of the problem is proposed to address the AMR problem, considering the food chain as a paramount factor for reducing and controlling resistance transmissions (European Commission, 2017; World Health Organization, 2018). Therefore, changes are required primarily in animal and plant production, by applying Good Agricultural Practices (GAP), and later in the animal food industry in the way of production, storage, processing, and distribution of foods of animal origin. FPA-human transmission of AMR increases the pressure of a reduced antimicrobial use in those animals. Therefore, prudent antimicrobial use in animal husbandries and control procedures targeting all foods of animal origin throughout the processing are the main effective intervention strategies to prevent the transmission of resistant bacteria from foods to humans and vice versa (Muaz et al., 2018). Furthermore, efforts to prevent such a challenge should also be built on application of effective food safety management, including Good Manufacturing Practices (GMP) and Good Hygienic Practices (GHP) during production and between food industry workers carrying the resistant strains so that person-to-person spread of these pathogens in animal food sector can be reduced (Lee, 2003; Normanno et al., 2007).

The use of bioprotective cultures is also proposed as a sustainable alternative to antibiotics in livestock production (Castellano et al., 2017). The antagonistic effect of LAB, as biopreservatives, is mainly due to a decrease of pH values in foods as well as the antibacterial activity of organic acids or peptides (bacteriocins) and bacteriocin-like inhibitory substances (BLISs), which can be exploited against foodborne pathogens (Gálvez et al., 2010). However, special caution should be taken to minimize the potential role of LAB as carriers or vectors of transferable AMR genes in ecofood systems. Therefore, the selection of the bacterial species that are intentionally added to foods should be based on Generally Recognized as Safe (GRAS) principle and supported by a thorough study of their biochemical and genetic characteristics in order to determine the presence of AMR genes or potential to act as carriers.

the strategies designed to combat this growing threat of MDR pathogens, face a particular challenge as there is a rapid dissemination of resistance genes between bacteria. Preventing the dissemination of antibiotic resistance genes can only be achieved with the continuous awareness of people working in agriculture and food sectors, and sustainable hygiene and sanitary practices (Centers for Disease Control, 2013).

## Concluding Remarks

Antibiotics are powerful drugs that are usually safe in treating microbial diseases in FPA for improving the health and welfare of animals; however, the indiscriminate use of antibiotics can lead to develop antibiotic resistance in animal microbiota. Due to the linkage between antibiotic use in FPA and the occurrence of antibiotic resistance diseases in humans *via* food chain, a judicious strategy regarding the use of antibiotics under only veterinary supervision in FPA should be promoted and reinforced. Rather than an “AMR amplifier,” we suggest that the food chain could act as a “resistances’ modulator” to reduce the incidence of resistant microorganisms by properly controlling food process parameters. Nevertheless, further research is needed to determine the further research is needed to determine both the mechanisms involved in the transmission of resistance and the food processing and storage conditions that are significant in their mitigation. Then, on this basis, robust control programs might be designed and put in place for the prevention of dissemination of the AMR genes between the bacteria along the food chain. The scientific basis to develop such mitigation strategies should be underpinned by a MRA scheme, whereby risk factors and transmission routes can be identified based on the combination of genomic data and predictive microbiology outcomes.

## Author Contributions

All authors listed have made a substantial, direct and intellectual contribution to the work, and approved it for publication.

### Conflict of Interest Statement

The authors declare that the research was conducted in the absence of any commercial or financial relationships that could be construed as a potential conflict of interest.
